# Ultrasound-intensified wet-heating induced Maillard reaction between hemp cake protein and maltodextrin: Structural characterisation, techno-functional and antioxidant properties

**DOI:** 10.1016/j.ultsonch.2025.107536

**Published:** 2025-08-27

**Authors:** Marzieh Sadeghi, Mohammad Mousavi, Mohammad Saeid Yarmand, Seyed Alireza Salami, Zahra Sarlak, Ehsan Parandi

**Affiliations:** a Department of Food Science and Engineering, University of Tehran, Iran; b Department of Horticultural Sciences, Faculty of Agriculture, University of Tehran, Karaj, Iran; c Research Center of Oil and Fats, Health Technology Institute, Kermanshah University of Medical Science, Kermanshah, Iran

**Keywords:** By-product, Plant protein, Glycation, Process Intensification, Emulsion

## Abstract

In this research, hemp protein isolate (HPI) was obtained from hemp cake using isoelectric precipitation and conjugated with maltodextrin through the Maillard reaction using wet-heating alone and combined with ultrasound to enhance its structural, functional properties (including solubility, emulsifying, foaming properties and water/oil holding capcity), and antioxidant properties. Covalent bonds between HPI and maltodextrin were confirmed using methods like SDS-PAGE, FTIR, XRD, fluorescence spectroscopy, secondary structure analysis, amino acid profile, and glycation degree (DG) measurement. Ultrasound significantly accelerated glycation, reaching a DG of 25.06 % in just 60 min, while reducing melanoidin formation, whereas wet-heating required 24 h to achieve a similar level of DG. Structural analysis revealed that wet-heating resulted in greater structural modifications due to its longer reaction time. These changes included a decrease in α-helix and β-sheet structures, higher surface hydrophobicity and zeta potential, and more noticeable microstructural changes. Techno-functional assessments revealed that wet-heating conjugates exhibited superior solubility (up to 95.77 %), emulsifying (emulsion stability index up to 48.67 %), and foaming (foaming capacity up to 107.14 %) properties, and antioxidant properties (71.95 % of ABTS), compared to ultrasound-assisted conjugates. This study highlights how ultrasound can intensify the wet-heating glycation of proteins to modify their structure, consequently improving their techno-functional properties to provide a novel plant-based protein ingredient for various food applications.

## Graphical abstract

**Figure:**
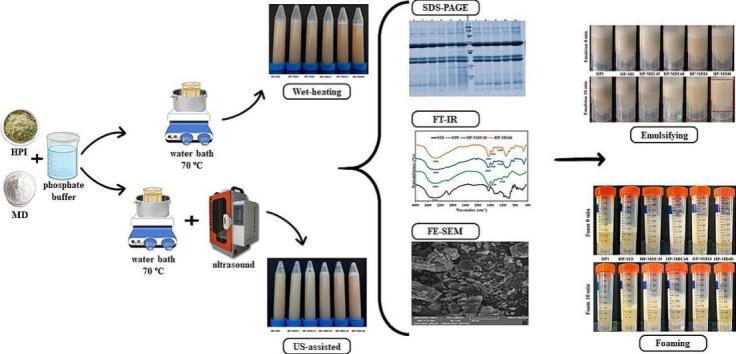

## 1 Introduction

With the global population increasing, the demand for sustainable and efficient protein sources is rising markedly. The manufacturing of animal-derived proteins poses considerable hurdles, including elevated economic costs, major environmental repercussions such as climate change, depletion of freshwater resources, loss of biodiversity, and related human health hazards [1]. As a result, plant-based proteins have attracted significant attention for their superior nutritional value, rendering them a sustainable and economical alternative [2]. Hemp protein is an excellent nutritional source, known for its well-balanced amino acid profile. This protein includes all essential amino acids, along with significant arginine and glutamic acid levels. In addition, hemp protein has an impressive digestibility rate of 88 %–91 %, significantly higher than soy protein's 71 % [3]. Despite its nutritional potential, hemp protein’s application remains limited due to its suboptimal techno-functional properties, including low solubility and weak emulsifying ability [4]. Considering the inherent connection between protein structure and its techno-functional properties, targeted modifications can enhance the techno-functionality of protein and expand its potential applications in various foods.

Protein modification can be achieved through various methods, including physical techniques (e.g., high-intensity ultrasound and high-pressure processing), chemical processes (e.g., glycation, phosphorylation, and acetylation), and biochemical approaches (e.g., oxidative enzymes such as oxidase and transglutaminase) [5]. Among these methods, chemical techniques provide notable effectiveness and relevance. However, many chemical modifications have drawbacks, including excessive reactions and the formation of toxic byproducts, which can lead to the presence of uncontrolled hazardous substances in the modified protein products. In contrast, glycosylation emerges as one of the most promising methods for enhancing the functional properties of natural proteins due to its moderate reaction process, stable modified products, and lack of hazardous byproducts. The glycation reaction combines the functional properties of native proteins and polysaccharides, thereby broadening the applications of native proteins in food processing [6].

This reaction is based on the Maillard reaction (MR), which occurs between the ε-amino groups of proteins and the carbonyl groups of reducing sugars. MR is a spontaneous reaction that takes place under precisely controlled conditions of temperature, pH, time, and humidity, without requiring the addition of chemicals [2,7]. Studies have demonstrated that protein glycation effectively enhances emulsifying properties, solubility, antioxidant activity, and antibacterial properties [8]. Traditionally, protein-carbohydrate bond formation can occur through either dry-heating or wet-heating techniques. The dry-heating approach requires more time and poses challenges in tracking the reaction's progress. In contrast, the wet-heating method is more efficient, enabling faster reaction completion and making it more suitable for industrial applications [9,10]. Nevertheless, the aforementioned thermal procedures for protein-carbohydrate conjugation are frequently time-consuming and may result in the generation of undesired browning chemicals, such as melanoidins. Consequently, it is important to utilise cutting-edge innovations that improve reaction kinetics and offer superior control over the degree of browning [11].

In recent years, ultrasound technology has gained attention in the food industry for its ability to enhance food properties and facilitate product modification. This technology is a fast and reliable approach to enhancing food quality, allowing for the creation of products with improved functional and sensory attributes. Ultrasound waves accelerate chemical reactions and enhance diffusion processes through acoustic cavitation [12]. Acoustic cavitation involves the formation, growth, and violent collapse of microbubbles in a liquid medium. This process generates localized regions of high temperature and pressure, intense shear forces, and microstreaming effects [13]. In comparison to conventional wet heating alone, ultrasound technology enhances the unfolding of protein structures and the rearrangement of peptide chains [14]. This exposes previously hidden free amino groups within secondary and tertiary structures to reaction. The increased accessibility to reactive sites, combined with enhanced molecular motion and microstreaming caused by cavitation, raises the collision frequency between reactive molecules, significantly accelerating the MR rate [13].

To the best our knowledge, the application of US-intensified wet-heating MR using maltodextrin (MD) to modify HPI remains unexplored. Current research is the first study to examine this specific approach and its effects on the structural, techno-functional, and antioxidant properties of HPI. Our findings provide mechanistic insights into how ultrasonic cavitation intensifies glycation and improves techno-functional properties of protein, offering a novel strategy to expand the application of HPI in food systems.

## 2 Material and methods

### 2.1 Materials

Hemp meal was purchased from a local oil factory (Karaj, Iran). MD (92 % *w/w*, DE16) was prepared by Zar Company (Karaj, Iran). Orthophthalaldehyde (OPA) and Ellman's reagent were purchased from Sigma-Aldrich (USA). β-mercaptoethanol, Sodium tetraborate decahydrate, Sodium dihydrogen phosphate, dibasic sodium phosphate, and all other chemicals were purchased from Merck (Germany).

### 2.2 Extraction of HPI

**Fig. 1:**
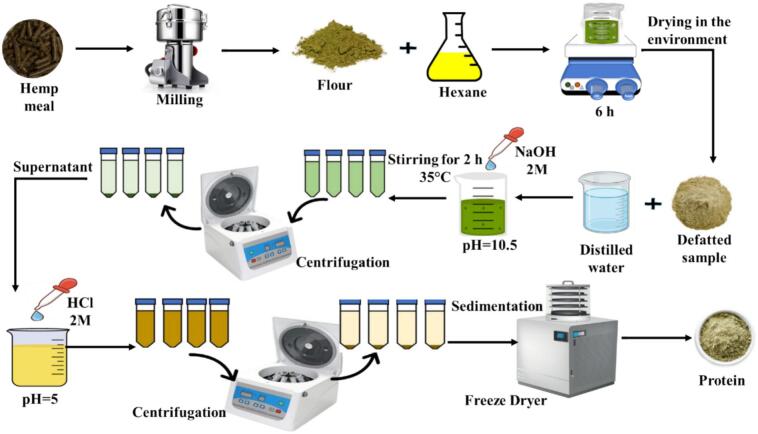
Schematic of HPI extraction procedure.

The meal was ground and passed through a 50-mesh sieve. The residual oil was extracted using 98 % n-hexane (meal-to-n-hexane ratio = 1:3 *w/v*) under continuous stirring for 6 h. The n-hexane was subsequently separated, and the precipitate was allowed to dry at room temperature. The defatted powder was dispersed in distilled water at a 1:10 ratio (*w/v*), and the pH was adjusted to 10.5 using 2 N NaOH. After stirring at 30–35 °C for 2 h, the suspension underwent centrifugation at 12,000*g* for 20 min. The supernatant's pH was set to 5 with 2 N HCl, followed by centrifugation at 12,000*g* for 20 min. The precipitate was washed two times with distilled water to enhance protein purity, and then it was centrifuged at 12,000*g* for 20 min. The collected pellet was resuspended in distilled water, adjusted to pH 7 using 0.5 N NaOH, and freeze-dried. The HPI powder was stored in the refrigerator for further analysis (Fig. 1) [15].

### 2.3 Chemical composition determination of HPI

According to AOAC 934.0, 923.03, and 920.39, the amounts of moisture, ash, and lipids were calculated, respectively. Based on the Kjeldahl method, the protein content in the hemp meal powder and the HPI was assessed according to the AOAC 979.09 standard (N × 6.25). Carbohydrate content was calculated by subtracting total protein, moisture, fat, and ash from 100.

### 2.4 Maillard conjugates preparation

Solutions of 2 % (*w/v*) HPI and MD were dissolved in phosphate buffer (0.1 M, pH = 8). Then, 0.2 % Sodium azide was added to each solution to inhibit microbial growth. The solutions were individually stirred for 5 h at ambient temperature and stored overnight in a refrigerator. The solutions were blended in a 1:2 (*w/w*) ratio of HPI to MD and stirred for 24 h until fully hydrated. The HPI–MD suspension's pH was regulated to 8 using 2 N NaOH. The suspension was then treated using the following two methods to initiate the MR [16].

#### 2.4.1 Wet-heating process

The HPI–MD suspension (400 mL) was incubated in a water bath at 70°C for 3 h (HP-MD3), 6 h (HP-MD6), 12 h (HP-MD12), 24 h (HP-MD24), and 48 h (HP-MD48). Finally, the samples were transferred to an ice-water bath to terminate the reaction (1).

#### 2.4.2 Ultrasound-intensified process

**Fig. 2:**
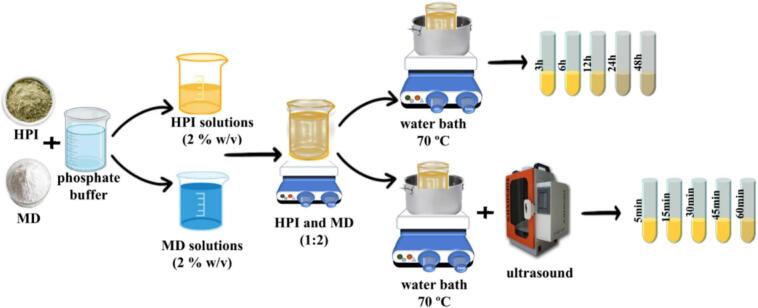
Schematic of the preparation of Maillard conjugates using wet-heating and ultrasound-intensified wet-heating methods.

The HPI–MD suspension (400 ml) was sonicated using an ultrasonic equipment (UHP-400, Topsonic, Iran) equipped with a horn-type probe (12 mm). Ultrasonic treatment was performed at a frequency of 20 kHz with a power output of 200 W, applying a 50 % duty cycle (2 s on, 2 s off). This corresponds to a power density of 10 W/mL. During sonication, samples were incubated in a 70°C water bath for 5 min (HP-MDU5), 15 min (HP-MDU15), 30 min (HP-MDU30), 45 min (HP-MDU45), and 60 min (HP-MDU60). Afterward, to terminate the reaction, the suspension was placed in an ice-water bath [17]. Finally, after freeze-drying, the samples were stored at 4°C for later use (Fig. 2).

### 2.5 Conjugates characterization

#### 2.5.1 Degree of glycation (DG)

The preparation of the OPA reagent involved dissolving 40 mg of OPA in 1 ml of methanol, followed by mixing with 50 ml of 0.1 M borax solution, 5 ml of 20 % (w/v) sodium dodecyl sulfate, and 100 μl of β-mercaptoethanol, and then adjusting the total volume to 50 ml with distilled water. After mixing 200 μl of the sample with 4 ml of the OPA reagent, the mixture was centrifuged at 4000 g for 20 min. The absorbance of the samples was measured at 340 nm using a UV–Vis spectrophotometer [18].

(1) $$DG\left(\%\right)=\frac{A_{B}-A_{T}}{A_{B}}\times 100$$

A_B_ is the absorbance of blank and A_T_ represents conjugate samples.

#### 2.5.2 Amadori and melanoidin compounds

In order to study the formation of intermediates (Amadori compounds) and advanced products (melanoidin compounds) during the MR, a UV–Vis spectrophotometer was employed to measure the UV absorption of the samples at 304 nm and 420 nm. For the measurement of Amadori compounds, solutions were prepared with a concentration of 10 mg/ml, while melanoidin compounds were prepared at 30 mg/ml using distilled water. After mixing at ambient temperature, the solutions were subjected to centrifugation at 4000*g* for 20 min. Finally, the supernatant's absorbance was recorded at the relevant wavelengths [19].

#### 2.5.3 SDS-PAGE

A 5 % stacking gel and a 12 % separating gel with 0.1 % SDS were utilized. Powder samples were dissolved in 1:2 (*w/v*) Laemmli buffer. Following a 5 min heating of a boiling water bath, the solution was centrifuged at 10,000*g* for 10 min. A 10 μl aliquot of the supernatant was added into the gel slots, and electrophoresis was carried out under constant conditions at 200 V for 4 h. The gel was immersed in Coomassie Brilliant Blue (R250) stain for 3 h and then destained using a solution composed of water, methanol, and acetic acid for 6 h [20].

#### 2.5.4 Amino acid composition

Treated and untreated protein samples were hydrolyzed with 6 M hydrochloric acid at 110 °C for 12 h. The hydrochloric acid was removed by transferring the samples to a 10 ml beaker, followed by placing them in a vacuum desiccator. Once dried, the samples were dissolved in 1 ml of 0.02 M hydrochloric acid and stored at 4 °C. The analysis of amino acid compounds was performed using an automated amino acid analyzer (Sykam GmbH, Germany). The amino acid composition was expressed in grams per 100 g of protein [21,22]

#### 2.5.5 FTIR spectroscopy

The freeze-dried HPI, MD, and conjugate samples were mixed with KBr in a 1:100 wt ratio and then pressed into semi-transparent tablets. An IR spectrometer (Thermo Fisher, USA) was used to record each sample in the frequency range of 400–4000 cm^−1^ [23,24].

#### 2.5.6 Circular dichroism (CD) spectroscopy

Protein secondary structure changes were investigated using Far-UV CD spectroscopy (Jasco Corp, Japan). The protein solution, diluted to a concentration of 1 mg/ml with 0.1 M phosphate buffer at pH 7.0, was scanned between 190 and 250 nm at 25 °C using a bandwidth of 1 nm. The proportions of secondary structures, including α-helix, β-sheet, β-turn, and random coil, were quantified using the CDPro software package with the SELCON3 algorithm [22,25].

#### 2.5.7 Fluorescence spectroscopy

Solutions with a concentration of 1 mg/ml were prepared using protein samples and conjugates in a phosphate buffer (0.05 M, pH 7). The fluorescence and emission spectra were recorded using a spectrometer (Shimadzu Corp., Kyoto, Japan), with an excitation wavelength of 280 nm, and the emission spectrum scanned over the range of 300–500 nm at a scan rate of 10 nm/s [22,26].

#### 2.5.8 X-ray diffraction (XRD)

X-ray diffraction (STAD IP, Japan) was conducted at 40 kV and 30 mA using Cu Kα radiation. The analysis was performed over a 2θ range of 5–80° with a scan speed of 2°/min [27,28].

#### 2.5.9 FE-SEM

The samples were placed on adhesive-coated holders and coated with approximately 10 nm of gold using ion sputtering equipment. The surface morphology of the samples was then observed at different magnifications and 15 kV in secondary electron mode using an FE-SEM (TESCAN MIRA3, Czech Republic) [29,30].

#### 2.5.10 Surface hydrophobicity (H_0_)

The hydrophobicity (H_0_) was determined using the 8-anilino-1-naphthalene sulfonic acid (ANS) fluorescent probe, as described by Parandi, Mousavi [21]. Protein concentrations of 0.05, 0.1, 0.2, 0.5, and 1 mg/ml were used to prepare the samples. A volume of 5 ml from each solution was combined with 20 µl of ANS (0.01 M). After being stored in the dark for 20 min, the fluorescence intensity of the samples was measured as a function of protein concentration using a Fluorescence Spectrophotometer (F-7100), with an excitation wavelength of 390 nm and an emission wavelength of 470 nm.

#### 2.5.11 Free sulphydryl (−SH) group

The measurement of free SH groups was carried out according to the procedure described by Pham, Wang [31]. The test was conducted using a Tris-HCl buffer (50 mM, pH = 8), which included 1 mM EDTA. The samples (30 mg) were dissolved in 3 ml of Tris-HCl buffer with 8 M urea. The samples were then centrifuged at 4000*g* for 20 min. For the preparation of Ellman's reagent, 4 mg of 5, 5-dithiobis nitro-benzoic acid (DTNB) was dissolved in 1 ml of Tris-HCl buffer. 20 µl of Ellman's reagent was then mixed with 2 ml of the supernatant and incubated for 1 h at room temperature, followed by absorbance measurement at 412 nm. The amount of free SH group was determined using the equation below:

(2) $$SH\left(\mu mol/g\right)=\frac{73.53\times A_{412}\times D}{C}$$

A_412_ refers to the sample's absorbance at 412 nm, D is the dilution factor, and C represents the protein concentration of the samples (mg/ml).

#### 2.5.12 Zeta potential (ZP) determination

Samples at a concentration of 1 mg/ml were dissolved in deionized water, and their Zeta potential was determined using a Zetasizer Nano Series (Malvern Instruments, UK) [21].

### 2.6 Techno-functional properties

#### 2.6.1 Solubility

The solubility properties of HPI and its conjugates were assessed by dispersing the samples in distilled water (1 mg/ml protein) and adjusting the pH from 3 to 11. After mixing for 2 h, the samples were centrifuged at 4000*g* for 30 min. The protein concentration in the samples was determined using the Lowry method and calculated based on a bovine serum albumin standard curve. Solubility was then calculated using the equation below [32].

(3) $$\;Solubility\;\left(\%\right)=\frac{w_{2}}{w_{1}}\times 100$$

W_1_ is the amount of total protein before centrifugation (*g*) and W_2_ is the amount of soluble protein after centrifugation (*g*).

#### 2.6.2 Emulsification properties

To determine the emulsifying activity index (EAI) and emulsion stability index (ESI) of HPI and its conjugates, protein solutions (10 mg/ml) were prepared in sodium phosphate buffer (0.1 mM, pH = 7). The samples were agitated for 3 h and placed in the refrigerator overnight to allow complete hydration. Sunflower oil was then added to each solution at a 4:1 (*v/v*) ratio, and the mixture was homogenized using an Ultra-Turrax T25 homogenizer (IKA, Staufen, Germany) at 10,000 rpm for 3 min. At 0 and 10 min post-homogenization, 50 µl of the sample was collected from the bottom layer of the emulsion. This sample was then diluted 100-fold with 0.1 % sodium dodecyl sulfate (SDS), and absorbance was measured at 500 nm. The EAI and ESI of the protein samples were calculated using the following equations [22,33]:

(4) $$EAI\left(\frac{m^{2}}{g}\right)=\frac{2\times 2.303\times A_{0}\times DF}{C\times \Phi \times \theta \times 10000}$$

(5) $$ESI\;\left(min\right)=\frac{A_{0}\times \Delta t}{A_{0}-A_{10}}$$

A_0_ and A_10_ represent the absorbance values at 0 and 10 min, respectively. DF denotes the dilution factor, C refers to the protein concentration (g/ml), Φ is the optical path length (1 cm), and θ represents the oil phase ratio (0.2)

#### 2.6.3 Foaming properties

The foaming capacity (FC) and stability (FS) of HPI and its conjugates were assessed by preparing 15 ml of a 1 % (*w/v*) protein solution in sodium phosphate buffer (0.1 mM, pH 7). The prepared solution was then homogenized at 10,000 rpm for 3 min. At 0 and 30 min following homogenization, the volume of the samples was measured [22,34]:

(6) $$FC=\frac{V_{1}-V_{0}}{V_{0}}\times 100$$

(7) $$FS=\frac{V_{2}-V_{0}}{V_{0}}\times 100$$

V_0_ (15 ml) is the initial volume, V_1_ is the volume of the solution right after homogenization (0 min), and V_2_ is the volume recorded after 30 min.

#### 2.6.4 Water and oil holding capacity

For the determination of water holding capacity (WHC) and oil holding capacity (OHC), 10 % (*w/w*) solutions of HPI and its conjugates were prepared in pre-weighed falcon tubes. These solutions were vortexed for 2 min and then allowed to equilibrate at room temperature for 1 h. The samples were then centrifuged at 4000*g* for 30 min. After centrifugation, the supernatant was gently discarded, and the falcon tubes containing the protein precipitate were re-weighed. The water and oil holding capacities of the samples were then calculated using the formula below [19]:

(8) $$WHC/OHC=\frac{W_{2}-W_{1}}{W_{0}}$$

#### 2.6.5 Antioxidant characteristics

The ABTS − RS test begins with the preparation of the stock solution, which involves mixing equal volumes of 7 mM ABTS solution and 2.45 mM potassium persulfate solution and incubating the mixture in the dark for 12–16 h at room temperature. The stock solution was then diluted with distilled water until the absorbance at 734 nm reached 0.7 ± 0.02, as measured using a UV–Vis spectrophotometer. Next, 200 µl of each sample (2 mg/ml protein) was combined with 1800 µl of the diluted stock solution, and the absorbance was measured at 734 nm after incubating in the dark for 5–10 min [31].

(9) $$ABTS-RS\left(\%\right)=\frac{A_{C}-A_{S}}{A_{C}}\times 100$$

### 2.7 Statistical analysis

All experiments were conducted in triplicate. Using SPSS 24 statistics software, the average data were subjected to analysis of variance (ANOVA) and Duncan's test at the (p-value < 0.05) confidence level.

## 3 Result and discussion

### 3.1 Chemical composition

Table 1 displays the chemical composition data for HPI and hemp meal. Following protein extraction, the purity of HPI significantly increased from 40.22 % to 92.11 % (*p* < 0.05). Comparable results have been documented in studies Ding, Jiang [1], Pang, Yang [35], and Shen, Gao [36] where purities exceeding 90 % were achieved using the isoelectric precipitation method. The protein extraction and recovery efficiencies in the current study were 14.34 % and 32.83 %, respectively. Additionally, the levels of other chemical compounds in HPI were significantly reduced compared with hemp meal (*p* < 0.05), consistent with trends observed in previous research.

**Table 1:** Chemical composition of hemp meal and HPI.

Sample	Parameters (%)				
	Protein	Moisture	Fat	Ash	Carbohydrates
Hemp meal	40.22 ± 0.09^b^	6.67 ± 0.09^a^	9.30 ± 1.01^a^	10.84 ± 0.04^a^	32.97 ± 0.89^a^
HPI	92.11 ± 0.31^a^	1.94 ± 0.13^b^	2.20 ± 0.2^b^	2.13 ± 0.04^b^	1.62 ± 0.31^b^

Values are mean ± standard deviation of a triplicate experiment. Mean values across a column followed by different superscript letters are significantly different (*p* < 0.05).

### 3.2 Conjugate characterization

#### 3.2.1 Glycation degree

**Fig. 3:**
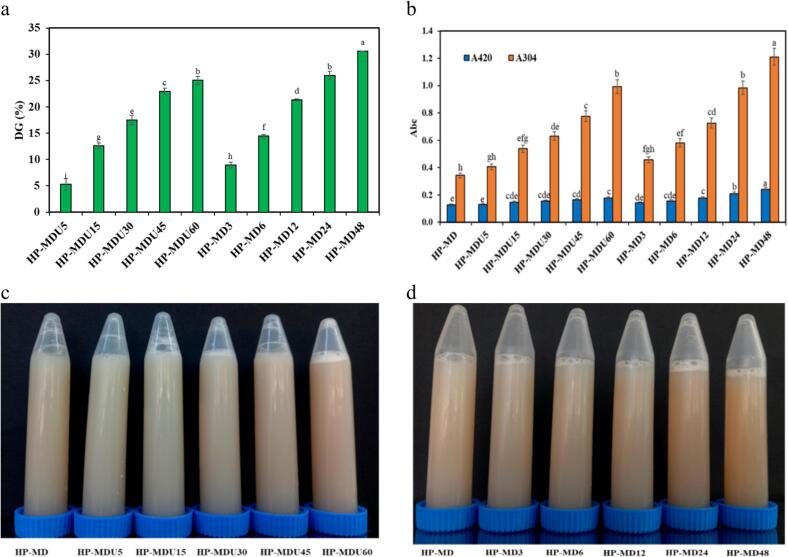
(a) Glycation degree% (DG) (b) absorbance at 420 and 304 nm of samples, (c) Visual alteration of ultrasound (d) and wet-heating treatments.

Fig. 3a illustrates the degree of glycation of the HPI and MD conjugates, which were produced using ultrasound-assisted and wet-heating methods at various reaction times. In both treatments, DG values increased significantly over time. Statistical analysis revealed that the DG value of the HP-MD48 treatment was significantly higher than those of the HP-MD12 and HP-MD24 treatments (*p* < 0.05). Similarly, among the ultrasound-treated samples, the HP-MDU60 treatment showed a significantly greater DG value compared to the HP-MDU30 and HP-MDU45 treatments (*p* < 0.05). These increases can be attributed to the structural unfolding of HPI during heat and cavitation treatments, which enhances the availability of free amino groups to interact with MD molecules [19,32]. The highest DG value was obtained for the HP-MD48 treatment, which reached 30.62 %. However, the DG of ultrasound-intensified treatments is comparable to that of wet-heating treatments, despite the shorter reaction time. For example, a DG of 25.96 % was obtained through wet-heating over 24 h, while a similar DG was achieved in approximately 60 min using ultrasound treatment. This indicates that achieving the same DG value takes longer with wet-heating than with ultrasound-assisted treatment [12]. Several studies have reported that ultrasound-assisted MR is an effective method for accelerating the cross-linking process between proteins and polysaccharides [9,37]. Ultrasound, as a sonic catalyst, forms cavitation bubbles that cause synergistic thermal, mechanical, and chemical effects during protein-polysaccharide interaction in the MR. The formation and sudden collapse of cavitation bubbles in the liquid induce shear stress and a temperature rise due to repeated positive pressure cycles, resulting in high turbulence conditions. In addition, ultrasound technology can enhance the speed of transfer processes, particularly heat and mass transfer, by generating a strong acoustic flow. Also, ultrasound can be beneficial by breaking down the protein's compact structure, which enhances the availability of free amino groups on the surface for reactions with polysaccharides [12,33]. It should be noted that the OPA method typically measures the loss of free primary amines; however, it does not necessarily confirm the formation of a covalent bond between HPI and MD in the treatments studied for both methods. Other factors, such as protein aggregation, may also reduce the availability of free amines. Therefore, although the OPA results indicate glycation, SDS-PAGE analysis was performed to complement our findings and confirm specific conjugation, ruling out other possible factors.

#### 3.2.2 Amadori and melanoidin compounds

The progress of MR was determined by measuring the absorbance of intermediate products (amadori compounds) at 304 nm and advanced products (melanoidins) at 420 nm. Fig. 3b indicates the amount of absorption of the wet-heating and ultrasound-assisted treatments. Significantly, the absorbance of the treatments at both wavelengths (304 and 420 nm) increased over time. This indicates that the bond between HPI and MD was formed through the MR, resulting in the formation of chromophores and a color change in the conjugates [37]. The conjugates' absorbance value at 304 nm is higher than that at 420 nm. This indicates mastery of the MR's initial stages under the conditions used. Comparable results were noted in the studies of Jiang, Zheng [10] On whey protein isolate and chia seed mucilage by wet-heating method, Naeini, Mousavi [25] on sesame protein-guar gum by wet-heating method, Zhang, Wang [38], on pea protein-maltodextrin by wet-heating method Sheng, Tang [39], on ovalbumin-pullulan by dry-heating method, and Stanic-Vucinic, Prodic [40] on β-lactoglobulin -glycoconjugates by ultrasound-assisted method. Fig. 3(c, d) indicates the visual color change of the mixture of protein and MD during MR. As is clear, the color of the treatment solutions has changed from light to brown over time. The degree of color change is more evident in wet-heating treatments than in ultrasound-assisted treatments, which is consistent with the absorbance readings of the treatments at a 420 nm wavelength. The extent of browning in the protein-polysaccharide mixture, aside from the MR and the formation of melanoidin compounds, may also result from the caramelization of sugar during the reaction [40]. This is particularly evident in the HP-MD24 and HP-MD48 treatments, which is a longer reaction time.

The reduced browning in ultrasound-assisted treatments may be due to the ultrasound-induced cavitation effects, which can inhibit polymerization of MR intermediates, thereby limiting melanoidin formation despite similar DG [17].

#### 3.2.3 SDS-PAGE

**Fig. 4:**
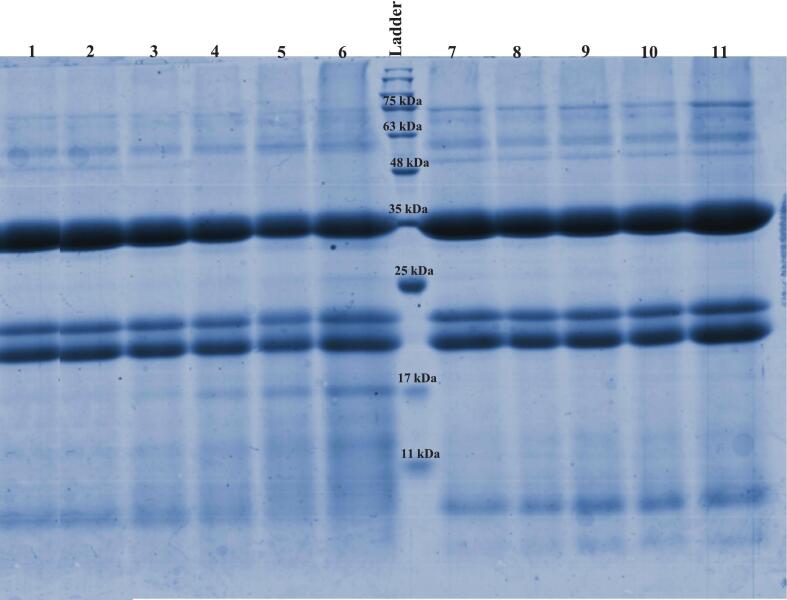
SDS-PAGE pattern of HPI and sample conjugates formed by wet-heating MR and Ultrasound-assisted MR at different times. Lane 1: (native HPI); lane 2: (HP-MD3); lane 3: (HP-MD6); lane 4: (HP-MD12); lane 5: (HP-MD24); lane 6: (HP-MD48); lane 7: (HP-MDU5); lane 8: (HP-MDU15); lane 9: (HP-MDU30); lane 10: (HP-MDU45); lane 11: (HP-MDU60).

SDS-PAGE is a reliable method for identifying covalent bonds formed between proteins and polysaccharides during MR [41]. Fig. 4 shows the electrophoretic profile of HPI and its conjugates formed by wet-heating and Ultrasound-assisted MR at different times. Based on previous studies, the main constituents of HPI are the proteins edestin (globulin) and albumin [33,42]. The band at about 55 kDa corresponds to globulin. Under reducing conditions, the disulfide bonds between the globulin subunits are broken. Consequently, the three clear bands observed at 19, 21, and 35 kDa represent the basic (19–21 kDa) and acidic (35 kDa) subunits of globulin [1]. A band observed at 9 kDa corresponds to albumin proteins [33]. After the MR, bands were observed at the upper section of the separation gel in the HP-MD48 and HP-MDU60 treatments. This observation is likely due to the formation of the HPI and MD copolymer, which decreases the mobility of the protein subunit because of its higher molecular weight [1,8,19,33]. Additionally, the appearance of a series of scattered bands on the acrylamide gel, particularly in the wet-heating conjugates, indicates the binding of MD molecules of varying molecular weights to HPI [8]. With increasing heating time, the intensity of the albumin band gradually diminished, and a new band at 17 kDa appeared in the wet-heating conjugates. In contrast, an increase in band intensity of approximately 55–75 kDa was observed in the Ultrasound-assisted conjugates. The changes in the protein bands indicate an increase in the protein's molecular weight subunits due to covalent bonding [1,33].

#### 3.2.4 Amino acid composition

The MR between the amino group of the protein and the reducing end carbonyl groups of the saccharides leads to a decrease in the content of various amino acids in the protein [21]. Table 2 shows the content of HPI, HP-MDU60, and HP-MD48 amino acids. The findings revealed that the proportion of hydrophilic amino acids (60.82–60.29 %) exceeded that of hydrophobic amino acids (39.18–39.71 %) across all three samples. However, following the creation of the bond between the protein and MD, the amount of hydrophilic amino acids decreased compared to HPI. A similar result was observed in the study by Qu, Zhang [9]. This demonstrates that the MR can modify the amino acid composition in the protein [10]. Compared to the amino acids in the samples conjugated with HPI, only the content of lysine was significantly decreased (*p* < 0.05), decreasing from 4.89 % in HPI to 4.12 % in HP-MDU60 and 3.79 % in HP-MD48. According to the statistical analysis, the lysine content in HP-MD48 was significantly lower than both HPI and HP-MDU60 (*p* < 0.05), indicating the progressive involvement of lysine in MR as the reaction proceeds. This demonstrates that the MR resulted in the covalent bonding of the amino groups in HPI with the carbonyl groups in MD. Therefore, it can be concluded that lysine was the only major site for the cross-linking reaction between MD and HPI. Although arginine also contains a reactive guanidino group and may theoretically participate in the MR [43], its content did not show any statistically significant change in our study, suggesting a limited involvement under the applied conditions. In addition, the levels of other amino acids also remained statistically unchanged (*p* > 0.05) across HPI and its conjugates. This can be explained by the loss of lysine amine groups due to participation in the reaction, along with the increased availability of other amine groups due to unfolding resulting from modification [44]. Alterations in the structure around lysine residues may influence the reaction efficiency between other amino groups and MD, as well as the amino group content of the protein [21,29,44].

**Table 2:** Amino acid profiles of HPI, HP-MDU60, and HP-MD48.

Amino acid	Concentration (g/100 g protein)		
	HPI	HP-MDU60	HP-MD48
*Hydrophilic*			
Arginine	10.31 ± 0.11^a^	10.39 ± 0.09^a^	10.39 ± 0.07^a^
Serin	5.28 ± 0.14^a^	5.33 ± 0.20^a^	5.34 ± 0.24^a^
Glycine	3.95 ± 0.36^a^	3.99 ± 0.27^a^	3.99 ± 0.21^a^
Lysine	4.89 ± 0.19^a^	4.12 ± 0.14^b^	3.79 ± 0.16^c^
Cysteine	1.17 ± 0.35^a^	1.21 ± 0.18^a^	1.24 ± 0.22^a^
Tyrosine	4.77 ± 0.23^a^	4.78 ± 0.12^a^	4.85 ± 0.15^a^
Histidine	2.87 ± 0.09^a^	2.91 ± 0.11^a^	2.95 ± 0.09^a^
Aspartic acid	10.41 ± 0.26^a^	10.48 ± 0.26^a^	10.49 ± 0.32^a^
Glutamic acid	17.17 ± 0.38^a^	17.26 ± 0.14^a^	17.25 ± 0.15^a^
Total	60.82 ± 0.24	60.47 ± 0.17	60.29 ± 0.18

*Hydrophobic*			
Valine	5.98 ± 0.13^a^	5.99 ± 0.19^a^	6.05 ± 022^a^
Threonine	4.63 ± 0.14^a^	4.68 ± 0.13^a^	4.68 ± 0.12^a^
Methionine	2.17 ± 0.07^a^	2.23 ± 0.14^a^	2.23 ± 0.12^a^
Alanine	5.5 ± 0.11^a^	5.57 ± 0.13^a^	5.58 ± 0.11^a^
Isoleucine	4.53 ± 0.18^a^	4.56 ± 0.21^a^	4.63 ± 0.26^a^
Proline	4.67 ± 0.05^a^	4.74 ± 0.09^a^	4.69 ± 0.05^a^
Leucine	6.83 ± 0.16^a^	6.87 ± 0.15^a^	6.91 ± 0.19^a^
Phenylalanine	4.87 ± 0.13^a^	4.89 ± 0.11^a^	4.94 ± 0.11^a^
Total	39.18 ± 0.12	39.53 ± 0.14	39.71 ± 0.15

Values are mean ± standard deviation of a triplicate experiment. Mean values across a column followed by different superscript letters are significantly different (*p* < 0.05).

#### 3.2.5 FTIR spectroscopy

**Fig. 5:**
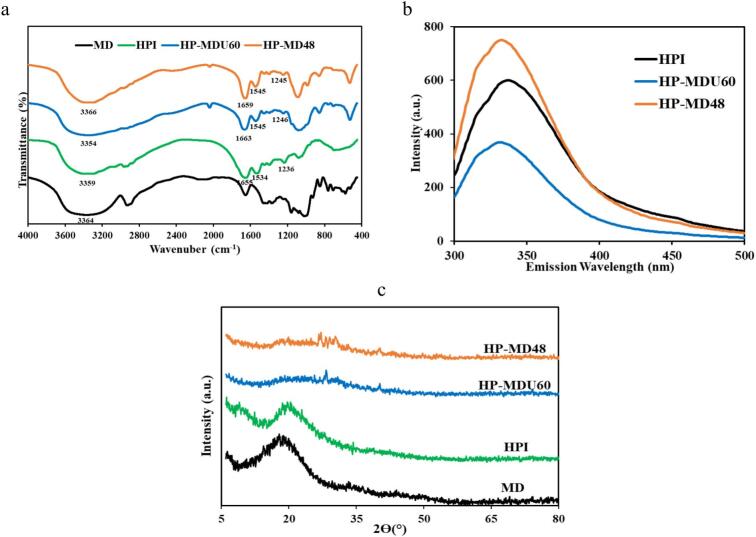
(a) FTIR spectra, (b) Fluorescence spectra, and (c) XRD patterns of samples.

FTIR can reveal details about the chemical structure of substances and the various bonds present due to its sensitivity toward specific functional groups [21]. Peaks 1655, 1534, and 1236 cm^−1^ in the HPI spectrum (Fig. 5a) correspond to the amide I (C=O stretching), amide II (C–N stretching and N–H bending), and amide III (C–N stretching and N–H bending) bands of the protein, respectively. As a result of the MR, changes have occurred in both the intensity and quantity of the amide I, II, and III peaks. Observed changes in the intensity and position of the amide band after reaction with MD indicate modifications in the protein's secondary structure, as well as the involvement of amino and carbonyl groups in covalent bonding during the MR. These changes correspond to the consumption of free NH_2_ and C=O groups, along with increased absorption peaks associated with Amadori products (C=O), Schiff bases (C=N), and pyrazine derivatives (C–N) [45]. For the HP-MDU60 sample, the absorption at 1663 cm^−1^ was higher than that in the HP-MD48 sample. This shows that the Ultrasound-intensified treatment can increase the active groups of proteins by breaking inter- and intramolecular hydrogen bonds. This causes the infrared spectrum to shift to higher wavenumbers [33]. Also, the absorptions of HP-MD conjugates around the region of 1080 cm^−1^ were stronger than those of HPI. This corresponds to strong stretching of the newly generated C–N bonds [9]. On the other hand, some new peaks appeared in both treatments around the region of 520–990 cm^−1^, which can be ascribed to C–O and C–C stretching vibrational modes and C–H bending modes. These are often referred to as “saccharide” bonds, resulting from covalent bond formation between HPI and MD [10]. These spectral changes indicate the formation of covalent bonds between protein and sugar moieties, which enhance the chemical stability and structural integrity of the conjugates. Such modifications are crucial for improving the functional properties of the resulting products.

#### 3.2.6 CD spectroscopy

CD spectroscopy is a useful tool for analyzing the secondary structure of proteins. The values of the second structures of HPI, HP-MDU60, and HP-MD48 are shown in Table. 3 The HPI secondary structure comprises 36.2 % α-helix, 32.5 % β-sheet, 15.9 % β-turn, and 15.4 % random coil. After MR, the α-helix and β-sheet structures decreased, while the β-turn and random coil structures increased compared to HPI. This shows that due to the formation of a bond between the HPI and the MD, the protein structure expands and changes from an ordered to a disordered form [5]. Since the α-helix and β-sheet structures play a crucial role in maintaining protein stability, the β-turns and random coil structures are responsible for their flexibility. It is expected that due to the increased flexibility of the protein, its emulsifying ability will improve [12,38]. In contrast to previous studies, which stated that the conjugates created with the Ultrasound-assisted method showed more specific changes in the second structure [2,9,33]. In this study, the wet-heating treatment indicated more changes compared to the Ultrasound-assisted treatment. Chen, Ma [8], reported similar results, noting that a conjugate of whey protein and gum acacia, created using the wet-heating method, exhibited more significant changes in its secondary structure compared to the Ultrasound-assisted conjugates.

**Table. 3:** . Secondary structure content of HPI, HP-MDU60, and HP-MD48.

Samples	Secondary structure (%)			
	α-helix	β-sheet	β-Turn	random coil
HPI	36.2 ± 0.12^a^	32.5 ± 0.21^a^	15.9 ± 0.14^c^	15.4 ± 0.28^c^
HP-MDU60	34.4 ± 0.11^b^	31.1 ± 0.23^b^	17.3 ± 0.19^b^	17.2 ± 0.30^b^
HP-MD48	28.5 ± 0.14^c^	28.2 ± 0.33^c^	19.5 ± 0.24^a^	23.8 ± 0.37^a^

Values are mean ± standard deviation of a triplicate experiment. Mean values across a column followed by different superscript letters are significantly different (*p* < 0.05).

#### 3.2.7 Fluorescence spectroscopy

Intrinsic fluorescence was utilized to study alterations in the protein's internal structure (tryptophan residues). Modifications in the protein’s tertiary structure can be deduced from changes in fluorescence emission maximum (λmax) and fluorescence intensity [33]. Under excitation at 280 nm, the λmax of HPI is 337 nm. This shows that the fluorescent amino acids of HPI (tryptophan, tyrosine, and phenylalanine) are in a “polar” environment [8]. As a result of MR, the λmax shifted to shorter wavelengths of 332 for HP-MD48 and 331 for HP-MDU60 (Fig. 5b). This shows that the hydrophobic environment surrounded the Trp residues in conjugates more than in HPI [46,47]. In the studies of Gao, Xu [48] on pea protein and glucose conjugates and Cheng, Mu [49] on rice protein and dextran conjugates, a blue-shifted λmax was observed. The fluorescence intensity of conjugates has also changed compared to the HPI, indicating that glycosylation changes the conformation of the protein [2]. On the other hand, HP-MD48 exhibited the highest fluorescence intensity compared to HPI and HP-MDU60, indicating that the protein has been denatured and its structure unfolded due to prolonged exposure to heat. This exposure reveals hydrophobic groups on the sample surface, which enhances fluorescence intensity [48]. The results of CD analysis confirmed that the protein structure in HP-MD48 was more open than that in HPI and HP-MDU60. In contrast, the decrease in fluorescence intensity of HP-MDU60 treatment compared to HPI can be explained by the protective effect of polysaccharide chains on Trp residues [26,37,46]. This trend indicates the varying impacts of different glycation methods on protein structure and the accessibility of tryptophan residues.

#### 3.2.8 XRD analysis

X-ray diffraction is often employed to study the crystalline structures of polysaccharides, proteins, and other macromolecular biopolymers. In XRD, sharp peaks indicate the crystalline regions of the sample, while broad peaks indicate the amorphous regions [27,50]. Fig. 5c shows XRD patterns of MD, HPI, HP-MDU60, and HP-MD48. The XRD pattern of MD and HPI has a semi-crystalline structure. The spectra of MD show a crystal peak at 2θ≈18.77° and the spectra of HPI show two crystal peaks at 8.99° (α-helix) and 19.65° (β-sheet). In the spectra of HP-MDU60 and HP-MD48, due to the interaction between HPI and MD, the X-ray diffraction peaks have become broader and smoother. This indicates a decrease in the crystallinity of both treated samples compared to the protein Parandi, Mousavi [21]. Li, Zhu [51], and Saatchi, Kiani [50] observed a similar result caused by the formation of a covalent bond between the protein and the polysaccharide. This bond causes the protein structure to unfold, facilitating polymer chains' mobility. Consequently, the crystal structures are reduced due to the interference of the protein chains.

#### 3.2.9 FE-SEM

**Fig. 6:**
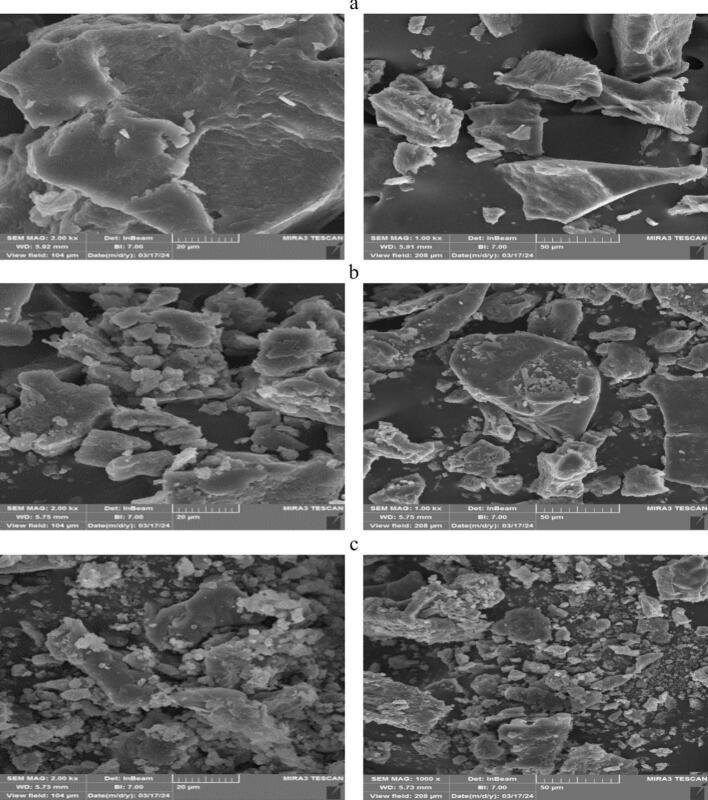
The surface structure of (a) HPI, (b) HP-MDU60, and (c) HP-MD48.

The study of the morphology of conjugates provides valuable information about their composition, particle size, and performance characteristics [33]. Fig. 6 shows the microstructures of the HPI, HP-MDU60, and HP-MD48. The HPI (Fig. 6a) features a flattened and smoothed surface structure, characterized by larger blocks and fewer connections between them. As can be seen, the surface of samples HP-MDU60 (Fig. 6b) and HP-MD48 (Fig. 6c) became rougher and more irregular. This observation confirms that a covalent bond has formed between HPI and MD [21]. These results are similar to those obtained by Zhong, Ma [29] on oat protein isolate-Pleurotus ostreatus β-glucan by dry-heating method and Wen, Zhang [2] on soy protein isolate-lentinan by ultrasound-assisted wet-heating method. It can be inferred that the binding of MD to the protein caused surface morphological changes in the native protein and inhibited its aggregation. These changes could improve the solubility of the conjugates compared to HPI [21].

#### 3.2.10 Surface hydrophobicity

**Fig. 7:**
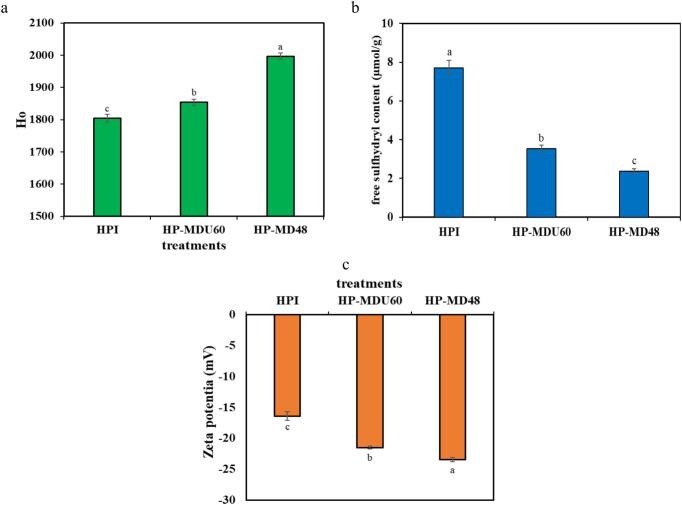
The (a) surface hydrophobicity, (b) free sulphydryl group samples, and (c) zeta potential.

Protein surface hydrophobicity reflects the presence of hydrophobic residues on the protein’s surface that interact with a polar environment [41]. In their natural state, most hydrophilic residues are situated on the protein’s surface, whereas hydrophobic residues are located in the interior [29]. Fig. 7a shows that the hydrophobicity values of the HP-MDU60 and HP-MD48 conjugates are higher than those of the HPI (*p* < 0.05). The covalent bond formed between the protein and MD during heat treatment or cavitation caused the protein structure to open up. This allowed a greater number of hydrophobic residues to migrate from the interior to the protein’s surface, thereby increasing its hydrophobicity [2]. In the studies of Ding, Jiang [1] on hemp protein isolate–pullulan, Liu, Yang [14] on ovalbumin–xylose, Wen, Zhang [2] on soy protein isolate–lentinan, and Chen, Ma [8] on whey protein isolate–gum acacia the hydrophobicity (H_0_) also increased after conjugation of the protein and polysaccharide. Additionally, relative to the HP-MDU60 conjugate, the HP-MD48h conjugate exhibited a higher H_0_ value, indicating that the protein structure has become more unfolded during wet-heating treatments than during ultrasound-assisted treatments [14]. The results of the CD analysis also confirm this.

#### 3.2.11 The amount of free sulfhydryl (−SH) groups

Disulfide bonds play a crucial role in maintaining the tertiary structure of proteins, and any modifications to these bonds can impact protein stability and functional properties [21,52]. During the MR, free −SH groups tend to undergo oxidation, leading to the formation of new disulfide bonds both within proteins and between proteins and polysaccharides. This process results in a measurable decrease in free–SH groups. Furthermore, the reaction of free–SH groups on the protein surface with MR products, such as Schiff bases and Amadori rearrangement products, also contributes to the reduction of free–SH content. Consequently, glycosylation significantly affects the sulfhydryl-disulfide exchange reactions (SH/S-S) [37]. Fig. 7b shows that the amount of −SH groups in both treatments decreased over time compared to HPI, suggesting the creation of a bond between HPI and MD. The concentration of −SH groups in wet-heating treatments is lower than in ultrasound-assisted treatments, which is consistent with the findings from DG. This may also be due to the breaking of disulfide bonds caused by ultrasonic cavitation, which results in a higher content of free SH. Liu, Xue [53], study of the conjugate of HPI and polyphenol aligns with this observation.

#### 3.2.12 Zeta potential

Zeta potential quantifies the surface charge of protein molecules and determines the extent of interparticle repulsion or attraction through the electrophoretic mobility technique [52]. The determination of surface charge can describe structural changes and the nature of electrostatic interactions on the surfaces of molecules, and can therefore be used to express the stability of colloidal dispersions [39,52]. Fig. 7c, shows that the zeta potential values for both the HP-MD48 (–23.5 mV) and HP-MDU60 (−21.5 mV) conjugates are significantly higher than those of the HPI (−16.8 mV) (*p* < 0.05). The free amine groups of the protein impart a positive charge to the protein at neutral and acidic pH due to the protonation of the amino groups. The formation of bonds between the HPI and the MD and the reduction of the free amine groups increases the negative charge of the protein [54,55]. On the other hand, the formation of covalent bonds caused the protein structure to unfold, leading to the exposure of negatively charged amino acids on the HPI surface [53]. A higher absolute value of the zeta potential indicates that the colloidal solutions derived from the conjugated samples exhibit greater stability than those from HPI.

### 3.3 Techno-functional properties

#### 3.3.1 Solubility

**Fig. 8:**
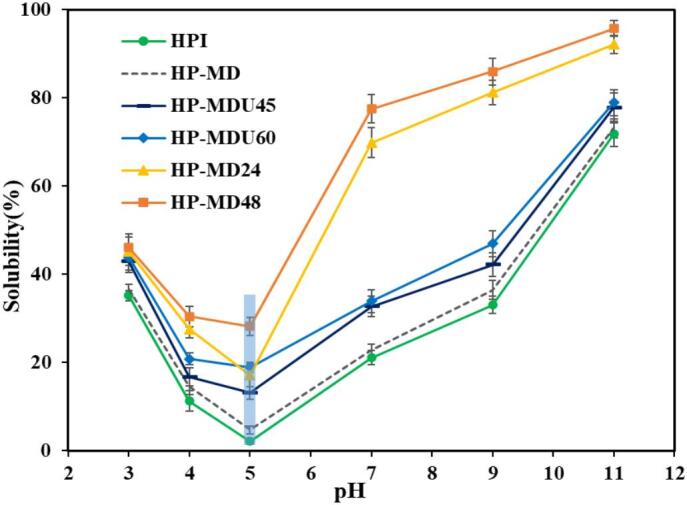
The solubility of HPI, HP-MD, and sample treatments.

Protein solubility is one of its most crucial functional properties, as it affects other characteristics like emulsification, foaming, and gelation [1]. Fig. 8 shows the water solubility of HPI, HP-MD, and HPI conjugates in the pH range of 3 to 11. In all samples, solubility was affected by pH, with the lowest solubility recorded at pH 5. This indicates that a pH of 5 corresponds to the isoelectric point of HPI and its conjugates [15]. During the extraction of hemp protein using the isoelectric method, mainly its globulin protein is extracted. This protein is spherical and has a rigid structure in which its hydrophilic groups are trapped inside [33,42]. This has led to poor solubility of HPI, at pH below 7 [42]. However, the solubility of HPI significantly increased after its covalent interaction with MD across all pH levels, especially at the isoelectric point. At this pH, the solubility of HPI rose from 2.23 % to 18.88 % in the HP-MDU60 treatment and to 28.17 % in the HP-MD48 treatment. The highest solubility observed at pH 11 was 95.77 % for the HP-MD48 treatment, while the solubility of HPI at the same pH was 71.82 %. This enhancement is likely due to the increased presence of hydrophilic groups (OH^–^) in the conjugates' structural formula, caused by the formation of a covalent bond with MD. This increase enhances the protein's affinity for interacting with water molecules [21]. FTIR analysis confirmed this by showing new absorption peaks related to C=O and C–N groups, indicating the presence of more polar functionalities that can interact with water. Furthermore, FE-SEM analysis revealed that the surface morphology of the conjugates became rougher and more irregular compared to the smooth, flat surface of native HPI. This change indicates the formation of covalent bonds between HPI and MD, leading to alterations in their surface structure. These structural changes, along with the steric hindrance caused by MD molecules, increased the surface contact with water and reduced protein aggregation, resulting in improved solubility of the conjugate samples compared to HPI [33]. Conjugates produced via wet-heating exhibited greater solubility, particularly at neutral and alkaline pH levels, compared to those prepared using the ultrasound-assisted heating method. This difference can be attributed to the results of the CD analysis. The results indicate that the protein structure was more unfolded during wet-heating treatments, which can convert insoluble protein aggregates into soluble forms, thereby increasing protein solubility [8]. Also, the results obtained from FE-SEM analysis demonstrated that the morphological surface of the HP-MD48 treatment had changed more than that of the HP-MDU60 treatment, which could improve the solubility [21]. The evidence obtained from FE-SEM and CD analyses can also explain the simultaneous observation of increased surface hydrophobicity and improved solubility. The unfolding of the protein structure exposes hydrophobic residues, resulting in increased surface hydrophobicity. However, the presence of hydrophilic groups arising from covalent bonding and the surface modifications prevent protein aggregation and enhance its interaction with water. Together, these factors contribute to the overall improvement in the solubility of the conjugate samples. The slightly higher solubility curve of HP-MD compared to HPI can be explained by the combination of MD with HPI and the introduction of additional hydrophilic groups [19].

#### 3.3.2 Emulsifying properties

**Fig. 9:**
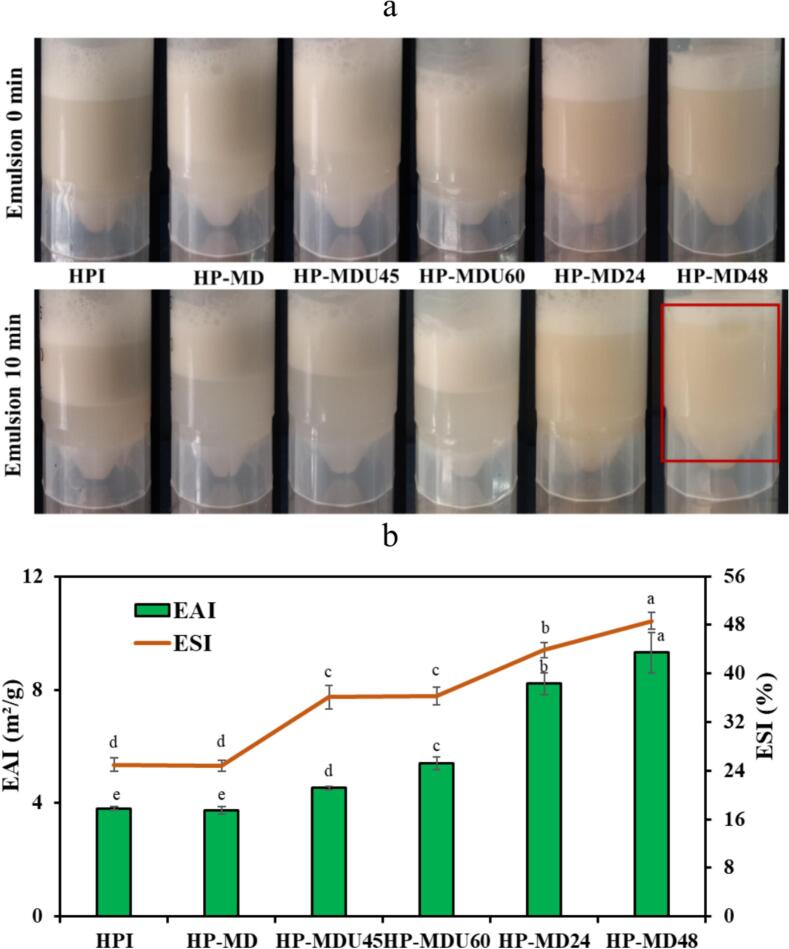
(a) Emulsion images, and (b) emulsion activity index (EAI) and emulsion stability index (ESI).

The EAI indicates the tension of surface protein molecules at the interface between oil and water and their emulsion-stabilizing ability, influenced by interactions between proteins and lipids. The ESI indicates the resistance of emulsions to separation and the retention of dispersion over time [35]. These two parameters are influenced by different factors. EAI is primarily associated with surface hydrophobicity, whereas ESI is typically determined by the surface charge of the proteins [56]. As shown in Fig. 9b, HPI has an EAI of 3.81 m^2^/g and an ESI of 25.1 %. The HPI and HP-MD mixtures showed no significant difference (*p* > 0.05) in ESI and EAI. Following glycation, a significant increase in the EAI of all conjugated samples was observed (*p* < 0.05) compared to the native protein. CD and SEM analyses revealed that the covalent bonding between HPI and MD expanded the protein’s secondary structure and increased surface irregularity. The reduction of ordered structures (α-helix and β-sheet) enhanced protein flexibility, while the increased surface roughness observed in FE-SEM images improved protein adsorption at the oil–water interface. These structural changes resulted in a decrease in surface tension and, consequently, an increase in EAI in the conjugated samples [44]. Notably, the EAI values for the HP-MD24 (8.21 m^2^/g) and HP-MD48 (9.31 m^2^/g) treatments are significantly higher than those for the HP-MDU45 (4.53 m^2^/g) and HP-MDU60 (5.40 m^2^/g) treatments. This increase can be attributed to improved solubility of the protein under wet-heating conditions, which enhances molecular mobility and facilitates better interaction at the oil–water interface, a key factor in ESI [12,17,44]. In addition, conjugate treatments demonstrated a significant increase in ESI compared to HPI. The formation of a bond between HPI and MD creates a thicker, more stable macromolecule around the oil droplets. This macromolecule prevents droplet aggregation and improves emulsion stability due to steric repulsion from the polysaccharide [57]. Similar to emulsifying activity, wet-heating treatments demonstrated greater stability than ultrasound-assisted treatments (*p* < 0.05). Emulsion stability is influenced by the surface charge of the protein. A higher zeta potential in the sample increases electrostatic repulsion, which helps prevent the aggregation of emulsion droplets [58]. The zeta potential measurements showed that the HP-MD48 treatment has a higher surface charge than the HP-MDU60 treatment. A higher zeta potential enhances electrostatic repulsion between droplets, which reduces aggregation and results in higher ESI values [52]. As a result, the emulsion stability of the HP-MD48 treatment (48.67 %) was higher than that of the HP-MDU60 treatment (32.36 %).

#### 3.3.3 Foaming properties

**Fig. 10:**
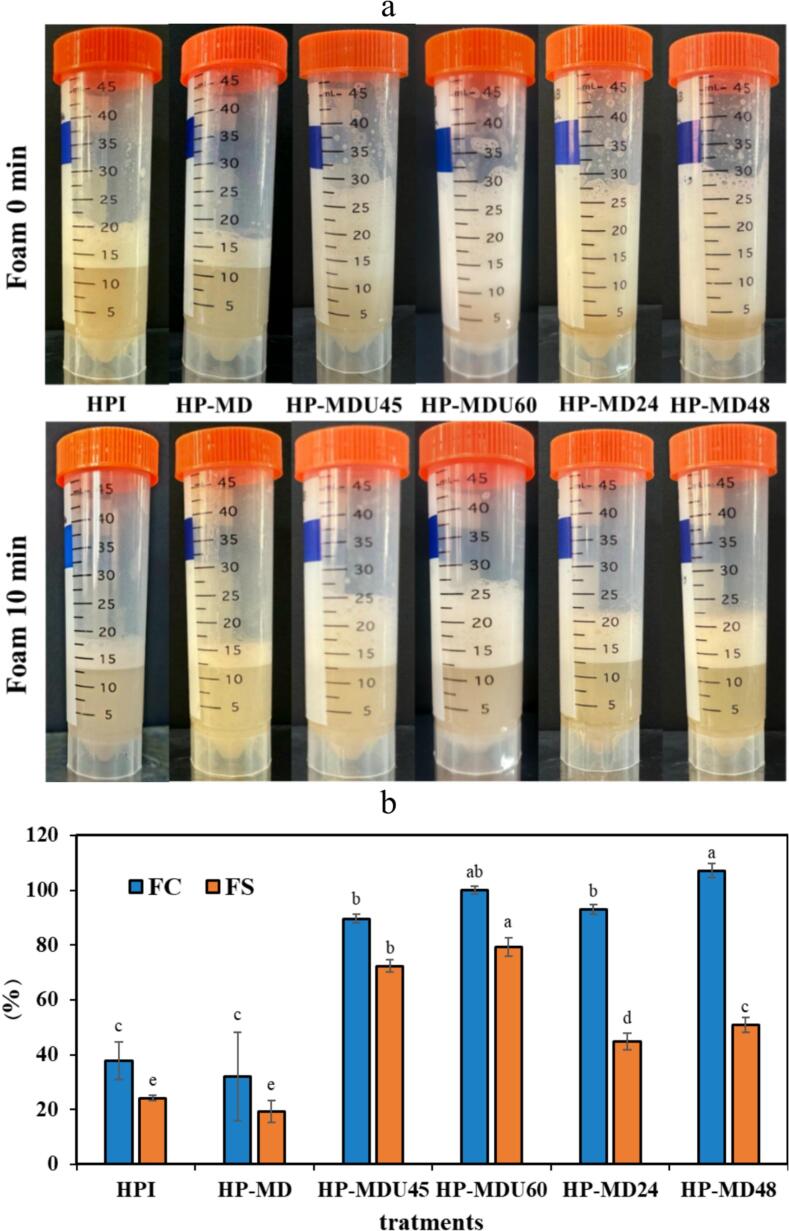
FC and FS, (a) images from real samples, and (b) results.

FC refers to the extent of surface area that a protein can generate, which is significantly influenced by its hydrophobicity. In contrast, FS refers to the foam's ability to withstand stress over time [59]. Fig. 10b displays the FC and FS values for HPI, HP-MD, and protein conjugates. There was no statistically significant difference in FC and FS between the HPI and HP-MD mixtures (*p* > 0.05). Following MR, a significant rise in FC and FS was observed in the treatments (*p* < 0.05). In the HP-MD48 treatment, FC and FS reached 107.14 % and 50.92 %, respectively, while in the HP-MDU60 treatment, they reached 100 % and 76.32 %, respectively. These results may be attributed to the regulation of the balance between protein hydrophobicity and hydrophilicity as well as the formation of conjugated compounds [60]. The attachment of MD molecules to the surface of HPI increased molecular flexibility and electrostatic repulsion between protein molecules. This structural modification enhanced foam stability, prolonging its retention [1]. Structural changes observed in the FTIR and CD spectra, as well as in the FE-SEM images, provide further evidence of these structural rearrangements that contribute to enhanced molecular flexibility and surface characteristics, which are crucial for the formation of a stable and resilient foam matrix.

#### 3.3.4 WHC and OHC

**Fig. 11:**
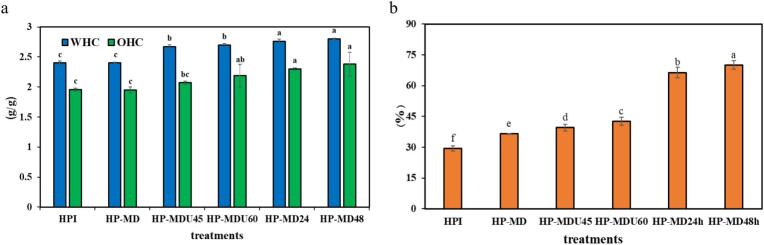
(a) WHC and OHC, and (b) antioxidant properties of samples.

WHC refers to the amount of water retained within the protein structure, reflecting its ability to limit water loss from its hydrated three-dimensional form after the application of centrifugal force. Similarly, OHC refers to a protein's ability to absorb and retain oil within its structure [42]. Fig. 11a displays the measured WHC and OHC levels for HPI and its conjugates. As shown, after conjugation, the WHC in all treatments increased significantly (*p* < 0.05) compared to HPI, rising from 2.4 *g/g* in HPI to 2.8 *g/g* in the HP-MD48 treatment and 2.7 *g/g* in the HP-MDU60 treatment. This increase is a result of the protein structure unfolding, which exposes its hydrophilic groups due to the MR. These hydrophilic groups enhance WHC by absorbing water molecules [19]. OHC followed a trend similar to WHC. The OHC increased from 1.95 *g/g* in the HPI to 2.38 *g/g* in the HP-MD48 treatment (*p* < 0.05). This increase can be attributed to the heightened surface hydrophobicity of the protein and the exposure of hydrophobic amino acids due to conjugation [1]. Proteins with higher surface hydrophobicity can absorb more fat, explaining why the OHC of the HP-MD48 treatment (2.38 *g/g*) increased more than that of the HP-MDU60 treatment (2.18 *g/g*). However, the two treatments showed no statistically significant difference (*p* > 0.05).

### 3.4 Antioxidant properties

The ABTS assay measures antioxidant activity by evaluating a sample's ability to neutralize free radicals via hydrogen atom transfer in an aqueous environment [61]. As shown in Fig. 11b, HPI exhibits an antioxidant activity of 31.4 %. Following the MR, the antioxidant activity of the treatments significantly increased compared to that of the native protein (*p* < 0.05). This enhancement is attributed to the unfolding of the protein structure, which exposes more hydroxyl and reducing groups, thereby increasing the availability of hydrogen atoms capable of scavenging ABTS free radicals [57]. Additionally, melanoidin compounds formed during the MR act as hydrogen donors, further enhancing antioxidant activity [25,62]. Accordingly, wet-heating treatments achieved the highest antioxidant activity, with 71.95 % for HP-MD48, whereas ultrasound-assisted treatments showed a lower increase, with 42.56 % for HP-MDU60. This difference can be explained by the longer reaction time in wet-heating, which promotes more extensive protein unfolding and melanoidin formation, resulting in superior radical scavenging capacity.

## Conclusion

This study demonstrates that HPI was successfully conjugated with MD using both wet-heating and ultrasound-intensified wet-heating methods to investigate their effects on the structure and kinetics of the MR. The findings indicate that ultrasound-intensified wet-heating treatment achieved a similar DG while significantly limiting melanoidin formation and completing the process in a fraction of the time required for wet-heating. In contrast, the HP-MD48 treatment induced more extensive structural modifications than the HP-MDU60 treatment, likely due to the longer reaction time facilitating more complete conjugation and protein modification. Both treatments significantly improved the techno-functional and antioxidant properties of the conjugates compared to the native protein, highlighting their potential as effective strategies for enhancing the functionality of food proteins. These findings highlight the efficiency of ultrasound-assisted glycation as a rapid and controlled strategy for enhancing protein functionality while minimizing thermal damage. However, the study is limited by its focus on fixed parameters. Future research should explore varying conditions, such as temperature, pressure, and different protein types, to optimize the process and broaden its applicability in food systems.

## Ethical statement

No animals were hurt in the making of this article.

## CRediT authorship contribution statement

**Marzieh Sadeghi:** Writing – original draft, Software, Methodology, Formal analysis. **Mohammad Mousavi:** Supervision, Project administration. **Mohammad Saeid Yarmand:** Conceptualization. **Seyed Alireza Salami:** Supervision. **Zahra Sarlak:** Writing – review & editing, Supervision, Data curation. **Ehsan Parandi:** Writing – review & editing, Validation, Supervision, Software, Project administration, Methodology, Formal analysis, Data curation.

## Declaration of competing interest

The authors declare that they have no known competing financial interests or personal relationships that could have appeared to influence the work reported in this paper.
